# Supplementary material for: Ag Nanostructures with Spikes on Adhesive Tape as a Flexible Sers-Active Substrate for In Situ Trace Detection of Pesticides on Fruit Skin
Source: Nanomaterials (Basel). 2019 Dec 9;9(12):1750. doi: 10.3390/nano9121750 (PMC6956212; doi:10.3390/nano9121750)
Supplement: Supplementary file 1 [file nanomaterials-09-01750-s001.pdf]

Table S1. Major characteristic peaks of Phosmet and Carbaryl and their corresponding vibrational modes [12, 25–27].

Pesticide	Observed Raman shift (cm^{-1})	Vibrational Assignment
Phosmet	606	C=O in-plane deformation vibration
	653	P=S in-plane deformation vibration
	1014	asymmetric stretching of P–O–C deformation
	1189	C–N in-plane deformation vibration
	1260	C–N stretching in S–CH_2–N
	1381	CH_3 in-plane deformation vibration
	1409	C–H out-of-plane deformation vibration
	1772	C=O stretching
Carbaryl	713	NCOC in-plane deformation vibration
	1380	symmetric ring vibration
	1582	C=C stretching in naphthalene ring

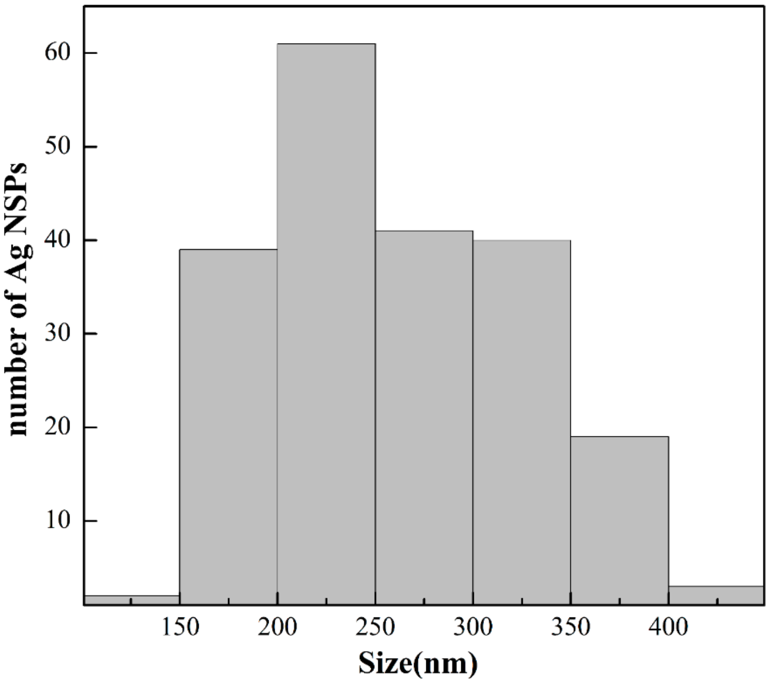

Figure S1. Size distribution of randomly selected 200 Ag NSPs showing an average size in terms of extended length of around 200-250 nm.
